# Intracellular Fibroblast Growth Factor 14: Emerging Risk Factor for Brain Disorders

**DOI:** 10.3389/fncel.2017.00103

**Published:** 2017-04-19

**Authors:** Jessica Di Re, Paul A. Wadsworth, Fernanda Laezza

**Affiliations:** ^1^Neuroscience Graduate Program, University of Texas Medical BranchGalveston, TX, USA; ^2^Department of Pharmacology and Toxicology, University of Texas Medical BranchGalveston, TX, USA; ^3^Biochemistry and Molecular Biology Graduate Program, The University of Texas Medical BranchGalveston, TX, USA; ^4^Mitchell Center for Neurodegenerative Diseases, The University of Texas Medical BranchGalveston, TX, USA; ^5^Center for Addiction Research, The University of Texas Medical BranchGalveston, TX, USA

**Keywords:** neuronal excitability, intracellular signaling, protein-protein interactions, biological psychiatry

## Abstract

The finely tuned regulation of neuronal firing relies on the integrity of ion channel macromolecular complexes. Minimal disturbances of these tightly regulated networks can lead to persistent maladaptive plasticity of brain circuitry. The intracellular fibroblast growth factor 14 (FGF14) belongs to the nexus of proteins interacting with voltage-gated Na+ (Na_v_) channels at the axonal initial segment. Through isoform-specific interactions with the intracellular C-terminal tail of neuronal Na_v_ channels (Na_v_1.1, Na_v_1.2, Na_v_1.6), FGF14 controls channel gating, axonal targeting and phosphorylation in neurons effecting excitability. FGF14 has been also involved in synaptic transmission, plasticity and neurogenesis in the cortico-mesolimbic circuit with cognitive and affective behavioral outcomes. In translational studies, interest in FGF14 continues to rise with a growing list of associative links to diseases of the cognitive and affective domains such as neurodegeneration, depression, anxiety, addictive behaviors and recently schizophrenia, suggesting its role as a converging node in the etiology of complex brain disorders. Yet, a full understanding of FGF14 function in neurons is far from being complete and likely to involve other functions unrelated to the direct regulation of Na_v_ channels. The goal of this Mini Review article is to provide a summary of studies on the emerging role of FGF14 in complex brain disorders.

## Introduction

In 2014, nearly one in four adults in the United States was diagnosed with a mental illness (National Institutes of Mental Health^1^). Treatment for many of these illnesses is hampered by limited efficacy of the medications and patient noncompliance due to intolerable side effects. To address this need, the National Institutes of Mental Health has launched an initiative to research these illnesses from all levels, ranging from genomic to behavioral. Dubbed the Research Domain Criteria (RDoC), this initiative proposes to complement top-down understanding of these diseases, beginning with human behavior, with bottom-up research by understanding the molecular and cellular causes of these disorders.

Because many neuropsychiatric disorders are associated with maladaptive plasticity and excitability, one area of importance within neurons is the axon initial segment (AIS), which serves as the action potential initiation site (Palmer and Stuart, 2006). This highly complex subcellular region contains a nexus of scaffolding and regulatory proteins that ensure proper targeting, clustering and function of the ion channels underlying the action potential (Ogawa and Rasband, 2008; Hsu et al., 2014). Neuropsychiatric disorders have been associated with many of these proteins, including ankyrin-G, α-spectrins, β-spectrins, neurofascin, contactin and intracellular fibroblast growth factors (iFGFs; Hsu et al., 2014). One such protein, fibroblast growth factor 14 (FGF14, also known as fibroblast homologous factor 4 or FHF4), is an iFGF that binds to voltage-gated Na^+^ (Na_v_) channels and promotes their localization to the proximal region of the axon, providing the fine-tuned regulation necessary for normal functioning (Lou et al., 2005; Laezza et al., 2007, 2009; Goetz et al., 2009; Wang et al., 2011, 2012; Wildburger et al., 2015; Ali et al., 2016; Bosch et al., 2016; Hsu et al., 2016; Pablo et al., 2016). Loss of functional FGF14 may change the biophysical properties of Na_v_ channels or alter their localization to the AIS, leading to changes in neuronal excitability (Goldfarb et al., 2007; Shakkottai et al., 2009; Bosch et al., 2015; Hsu et al., 2016). Recent findings also show that FGF14 regulates the function of voltage-gated K^+^ and Ca^2+^ channels, however none of these interactions are direct, and therefore might represent a different type of regulation from what has been described for Na_v_ channels (Yan et al., 2013; Pablo and Pitt, 2017).

Initially cloned on the basis of sequence similarity with other FGF members, FGF14 was first associated to a human disease with the F145S mutation causing spinocerebellar ataxia 27 (SCA27), a naturally occurring complex neurodegenerative disorder characterized by onset of ataxia in early adulthood and deficits in cognition, memory and behavior (Smallwood et al., 1996; van Swieten et al., 2003; Brusse et al., 2006). Genetic deletion of FGF14 in mice recapitulates some of these symptoms at the behavior and circuitry level (Wang et al., 2002; Wozniak et al., 2007).

Since then, FGF14 has been indicated by several linkage and genome wide association studies (GWAS) to be a putative risk factor for other neuropsychiatric diseases including depression, addiction and schizophrenia, as well as neurodegenerative diseases, such as Alzheimer’s Disease (Detera-Wadleigh et al., 1999; Park et al., 2004; Mulle et al., 2005; Need et al., 2009; Johnson et al., 2011; Verbeek et al., 2012; Singh and Rajeswari, 2015; Yang et al., 2015). These recent associations clearly indicate that the role of this gene in the CNS is yet to be fully understood. In the next paragraphs, we will summarize some of the most recent studies on FGF14 in animal models and human tissue.

## Historical Prospective: FGF14 as Voltage-Gated Na^+^ (Na_v_) Channel Interacting Protein

**Figure 1:**
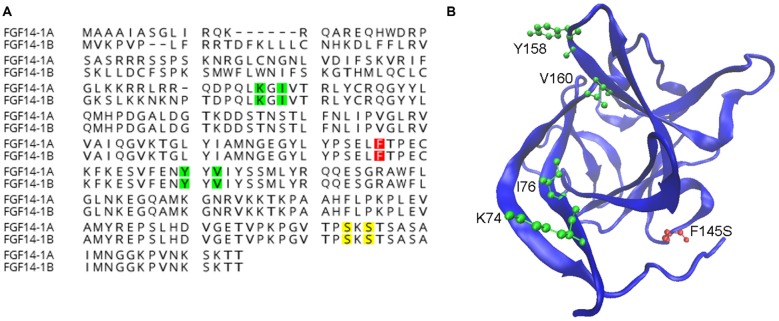
**Known functions of amino acids in fibroblast 14 (FGF14). (A)** Alignment of wild-type FGF14-1a and FGF14-1b protein sequences with highlighted residues representing surface hot-spots for FGF14:Na_v_1.6 interactions (green, Ali et al., 2016), casein kinase II (CK-2) phosphorylation sites (yellow, Hsu et al., 2016), and the spinocerebellar ataxia 27 (SCA27) F145S mutation site (red, Laezza et al., 2007). **(B)** An FGF14 homology model generated using the FGF13:Na_v_1.5 crystal structure (Protein Data Bank ID: 4DCK) as template, visualized using the visual molecular dynamic (VMD) software package (Humphrey et al., 1996). Surface hot-spots important for protein:protein interactions (green) and the SCA27 F145S mutation (red) are shown.

While iFGFs share a conserved core β-trefoil region with other FGFs their functions and distributions are distinct from canonical FGFs (Itoh and Ornitz, 2008). Canonical FGFs are normally secreted to activate FGF receptors on the cell surface, however iFGFs lack a secretory sequence, fail to activate or antagonize FGF receptors and are primarily found in the cytoplasm, nucleus or the AIS (Smallwood et al., 1996; Olsen et al., 2003; Ornitz and Itoh, 2015; Pablo et al., 2016). Initial discoveries using yeast-two-hybrid screening identified FGF12 and FGF13 as direct interactors of Na_v_ channels (Liu et al., 2003; Wittmack et al., 2004; Rush et al., 2006) and subsequent studies resulted in similar discoveries for the two isoforms of FGF14 (Lou et al., 2005; Laezza et al., 2007, 2009), aligned in the illustration of Figure 1A. To date, the evidence for direct interaction and functional modulation of Na_v_ channels by FGF14 ranges from crystal structure to biochemical in cell assays to animal models and includes recent identification of critical amino acid residues at the FGF14: Na_v_1.6 channel complex illustrated in Figure 1B (Lou et al., 2005; Laezza et al., 2007, 2009; Goetz et al., 2009; Ali et al., 2014, 2016; Hsu et al., 2016; Pablo et al., 2016).

The N-terminus of FGF14 is alternatively spliced into two isoforms: FGF14-1a and FGF14-1b. FGF14-1a shares sequence homology with FGF12-1a and FGF13-1a, while the amino terminus of FGF14-1b contains a unique 69 amino-acid sequence and is the more prevalent isoform in the CNS (Munoz-Sanjuan et al., 2000). Importantly, the interaction of FGF14 in cells that heterologously express individual Na_v_ isoforms shows that FGF14 is unique, as it results in very distinct isoform-specific Na+ current phenotypes that are dictated by the two FGF14 splice variants (Lou et al., 2005; Laezza et al., 2009; Ali et al., 2016). In primary hippocampal neurons, overexpression of FGF14-1b increases Na+ current density, causes a hyperpolarizing shift in the voltage-dependence of activation and a depolarizing shift in the voltage-dependence of inactivation, while the F145S, SCA27 dominant negative loss-of-function mutation causes opposite phenotypes, possibly by disrupting the function of wild-type FGF14 (Laezza et al., 2007). Neurons from *Fgf14*^−/−^ mice exhibit impaired excitability in both the hippocampus and cerebellum (Goldfarb et al., 2007; Shakkottai et al., 2009; Bosch et al., 2015; Hsu et al., 2016). The effect of FGF14 on Na_v_ channels and excitability is summarized in Table 1.

**Table 1:** **Effect of splice variants in heterologous systems and knockout animals**.

Isoforms	FGF14-1a (HEK-293)	FGF14-1b (HEK-cells)	FGF14-1b (Neuronal cell line)	FGF14-1b (Hippocampal neurons)	FGF14 Knockout (Granule neurons and CA1 hippocampal) neurons)
Na_v_1.1	Depolarizing shift in voltage dependence of activation, depolarizing shift in voltage dependence of inactivation (Lou et al., 2005)	Decreases in current density, depolarizing shift in voltage dependence of inactivation (Lou et al., 2005)	Decreases current density (Laezza et al., 2009)	-	-
Na_v_1.2	Depolarizing shift in steady state inactivation (Laezza et al., 2009)	-	Decreases current density, small depolarizing shift in steady-state inactivation (Laezza et al., 2009)	-	-
Na_v_1.5	Decreases current density, depolarizing shift in voltage dependence of inactivation (Lou et al., 2005)	Decreases current density, hyperpolarizing shift in inactivation (Lou et al., 2005)	-	-	-
Na_v_1.6	Depolarizing shift in steady-state inactivation, slower recovery from inactivation (Laezza et al., 2009)	-	Decreases current density, depolarizing shift in steady-state inactivation (Laezza et al., 2009)	-	-
Native Na_v_ channels	-	-	-	Increases current density, hyperpolarizing shift in voltage-dependence of activation, depolarizing shift in steady-state inactivation (Laezza et al., 2007)	Reduces evoked repetitive firing (Goldfarb et al., 2007; Hsu et al., 2016)

## FGF14 as Scaffold for Kinases

Recent studies have added a new dimension to FGF14, showing that its interaction with the Na_v_ channel is controlled by selective kinases (Shavkunov et al., 2012, 2013; Hsu et al., 2015, 2016). Initial studies using the luciferase complementation assay demonstrated that the FGF14:Na_v_1.6 complex formation is controlled by glycogen synthase kinase 3 (GSK; Shavkunov et al., 2013) and more recently by the GSK3 priming kinase, casein kinase II (CK2) which phosphorylates FGF14 at S228 and S230 (Hsu et al., 2016; Figure 1B). Inhibition of either GSK3 or CK2 is sufficient to disrupt the FGF14:Na_v_ channel complex formation with consequences for targeting of the two proteins to the AIS and for intrinsic excitability (Shavkunov et al., 2013; Hsu et al., 2016). It is possible that phosphorylation at these kinase specific sites that confers functional specificity to FGF14 contributing to regulation of other ion channels (i.e., voltage-gated K^+^ and Ca^2+^ channels).

More is known about the specific phosphorylation of FGF14 by GSK3 and CK2, however other kinases have been shown to affect FGF14:Na_v_1.6 interactions (Shavkunov et al., 2012; Hsu et al., 2015). Importantly, many kinases involved in tyrosine receptor kinase signaling are implicated in this regulation, including the mitogen activated protein kinase (MAPK), C-Jun N-terminal kinase (JNK; Hsu et al., 2015). JNK signaling is disrupted in insulin resistance associated with type-II diabetes and Alzheimer’s disease (Najem et al., 2016). Functional enrichment of single nucleotide polymorphisms (SNPs) in patients with type-II diabetes and Alzheimer’s disease shows that FGF14 is significantly overrepresented in these two diseases because of its phosphorylation by JNK (Hao et al., 2015). Changes in the mRNA expression of MAPK/JNK signaling proteins, including FGF14, are also significantly overrepresented in early-onset Alzheimer’s disease patients (Antonell et al., 2013). Taken together, these results indicate that the interaction between JNK and FGF14 might be an important area for future research in Alzheimer’s disease.

## FGF14 as Regulator of Excitatory and Inhibitory Synaptic Transmission

Numerous are the reports of the effect of genetic deletion of *Fgf14* on synaptic transmission. Studies in the cerebellum of *Fgf14*^−/−^ mice revealed decreased excitatory transmission from granule cells to Purkinje cells (parallel fibers, PF), a phenotype that is accompanied by reduced AMPA receptor-mediated excitatory postsynaptic currents and decreased expression of vesicular glutamate transporter 1, a specific presynaptic marker at PF-Purkinje neuron synapses (Tempia et al., 2015). Presynaptic changes in neurotransmitter release have been also reported at the Schaffer’s collaterals to CA1 synapses where deletion of *Fgf14* results in reduction in the ready-releasable pool of presynaptic glutamate and diminished expression of synaptobrevin, synaptophysin, syntaxin I (Xiao et al., 2007). Other changes in presynaptic function have been reported at inhibitory GABAergic terminals onto CA1 pyramidal cells of *Fgf14*^−/−^ mice, which exhibit reduced expression of glutamic acid decarboxylase 67 (GAD67) and vesicular GABA transporter (vGAT), presumably deriving from fast-spiking parvalbumin (PV) interneurons synapses (Alshammari T. K. et al., 2016). Additional studies in the same animal model identified selective loss of PV interneurons, reduced γ frequency oscillations and deficits in working memory (Alshammari T. K. et al., 2016). Collectively, these results recapitulate some endophenotypes of schizophrenia and are supported by human studies finding significant reduction and co-variation of *FGF14*, *PV*, *vGAT* and *GAD67* in post-mortem samples from schizophrenic patients compared to healthy control individuals (Alshammari T. K. et al., 2016). Whether all these changes at presynaptic glutamatergic and GABAergic terminals result from neuroadaptive responses to impaired firing or represent disruption of a separate function of the FGF14 protein remains to be determined. However, the evidence for genetic links between *vGAT*, *GAD67* and *FGF14* might argue for a “separate function” hypothesis of FGF14 that results from a control at the gene level.

## FGF14 Is Required for Synaptic Plasticity

Studies have also supported a role of FGF14 in synaptic plasticity in the hippocampus. *Fgf14*^−/−^ mice show impaired long-term potentiation (LTP) at the Schaffer’s collaterals to CA1 synapses, which is accompanied by decreased expression of synaptic vesicles docked at the active zone, and fewer miniature excitatory postsynaptic currents in primary hippocampal neurons (Xiao et al., 2007). Short-term plasticity is also impaired at these *Fgf14*^−/−^ terminals, at which repetitive stimuli causes significant synaptic fatigue, consistent with impaired presynaptic function (Xiao et al., 2007).

## FGF14 as Factor Required for Neurogenesis

Adult neurogenesis, or the proliferation, differentiation and integration of new neurons into existing brain circuitry has become an area of research interest in part due to its implication in the cognitive pathophysiology of several neuropsychiatric disorders, including Alzheimer’s disease, depression and schizophrenia (Ming and Song, 2005; Taupin, 2005, 2008; Reif et al., 2007; Johnson et al., 2009; Sun et al., 2011; Jun et al., 2012; Walton et al., 2012; Ouchi et al., 2013). It was recently found that FGF14 is required for the maturation of progenitor cells in the dentate gyrus of the hippocampus. *Fgf14*^−/−^ mice show impaired transition from late immature neuronal progenitor cells to mature neurons, which is accompanied by reduced paired-pulse facilitation at the perforant path to granule neurons in the dentate gyrus (Alshammari >M. A. et al., 2016). Overall, deletion of FGF14 results in an immature dentate gyrus, an endophenotype that corroborates a link between the gene and schizophrenia (Hagihara et al., 2013).

## FGF14 as An Associated Factor for Neuropsychiatric Disease

As many neuropsychiatric disorders are heterogeneous and complex, GWAS have become an important tool for sorting relevant genetic information from large patient populations. Numerous GWAS have reported SNPs in *FGF14* in the context of neuropsychiatric disorders (Table 2). Although all these SNPs are in the *FGF14* intronic region and thus their role on the protein expression and function are unclear, they might provide guidance for future investigations. A Brazilian pilot study on early onset/familial schizophrenia found a link between early-onset schizophrenia and *FGF14* (Gadelha et al., 2012). GWAS in German cohort found an association between *FGF14* and schizophrenia, which is corroborated by a linkage study of familial schizophrenia in Canadian families of Celtic or German descent (Brzustowicz et al., 1999; Need et al., 2009). Additionally, SNPs in *FGF14* have been associated with dependence on alcohol and illegal substances in humans, and a fine-mapping study found several SNPs to be associated with major depressive disorder in a study of Dutch twins (Drgon et al., 2011; Johnson et al., 2011; Verbeek et al., 2012). Furthermore, an *FGF14* SNP is associated with volumetric changes in the entorhinal cortex in AD patients (Yang et al., 2015). Overall, genetic variations in *FGF14* are linked to the pathophysiology of several neuropsychiatric disorders, a promising area for further research that is supported by studies in *Fgf14*^−/−^ preclinical models (Alshammari T. K. et al., 2016; Alshammari >M. A. et al., 2016).

**Table 2:** **Single nucleotide polymorphisms (SNPs) in FGF14 introns associated with neuropsychiatric disease**.

Intronic SNP ID	Associated disease	Citation
rs636674	Major depressive disorder	Verbeek et al. (2012)
rs1457315	Major depressive disorder	Verbeek et al. (2012)
rs4772439	Major depressive disorder	Verbeek et al. (2012)
rs7992504	Major depressive disorder	Verbeek et al. (2012)
rs9518615	Major depressive disorder	Verbeek et al. (2012)
rs9518638	Major depressive disorder	Verbeek et al. (2012)
rs9557792	Major depressive disorder	Verbeek et al. (2012)
rs128655694	Major depressive disorder	Verbeek et al. (2012)
rs17688345	Major depressive disorder	Verbeek et al. (2012)
rs35700852	Major depressive disorder	Verbeek et al. (2012)
rs4772445	Schizophrenia	Need et al. (2009)
rs9554852	Substance dependence	Drgon et al. (2011)
rs16959573	Substance dependence	Johnson et al. (2011)
rs17502818	Substance use	Johnson et al. (2011)
rs2476230	Antidepressant response	Hunter et al. (2013)
rs17502999	Entorhinal cortex volume change in Alzheimer’s disease	Yang et al. (2015)

## Conclusion

FGF14 plays a role in all fundamental properties of neurons: intrinsic firing, synaptic transmission of excitatory and inhibitory neurons and plasticity, while deletion of the gene leads to disruptive motor and cognitive behaviors. The role of FGF14 in humans is yet to be fully understood, but the emerging technologies for genome sequencing and protein characterization will provide potential opportunities for identifying new disease signatures associated with FGF14.

## Author Contributions

All of the authors have contributed substantially to the work. JDR and FL contributed to writing and editing the manuscript. PAW created the image and legend for Figure 1.

## Funding

This research was funded by National Institute of Mental Health: grant no. 1R01MH111107-01A1, R01 MH095995-A1 and the National Institutes of Health: grant no 5T32AG051131-02.

## Conflict of Interest Statement

The authors declare that the research was conducted in the absence of any commercial or financial relationships that could be construed as a potential conflict of interest.

## Footnotes

^1^https://www.nimh.nih.gov/health/statistics/index.shtml
